# Contrast in soil microbial metabolic functional diversity to fertilization and crop rotation under rhizosphere and non-rhizosphere in the coal gangue landfill reclamation area of Loess Hills

**DOI:** 10.1371/journal.pone.0229341

**Published:** 2020-03-09

**Authors:** Bian-hua Zhang, Jian-ping Hong, Qiang Zhang, Dong-sheng Jin, Chun-hua Gao

**Affiliations:** 1 College of Resources and Environment, Shanxi Agricultural University, Taigu, Shanxi, China; 2 Xinzhou Teachers University, Xinzhou, Shanxi, China; 3 Institute of Agricultural Environment and Resources, Shanxi Academy of Agricultural Sciences, Taiyuan, Shanxi, China; ICAR-National Rice Research Institute, INDIA

## Abstract

Very poor reclaimed soil quality and weak microbial activity occur in the reclamation area of a coal gangue landfill in the Loess Hills. The fourth and fifth years after farmland soil was reclaimed were studied, and the changes in and carbon source utilization characteristics of rhizosphere (R) and non-rhizosphere (S) soil microorganisms under organic and inorganic (OF), inorganic (F), and organic (O) fertilizer application and a control treatment (CK) in soybean (S) and maize (M) rotation systems were compared and analysed in Guljiao Tunlan, Shanxi Province, China. Biolog-EcoPlate technology was used to analyse the mechanism of soil characteristic change from the perspective of soil microbial metabolism function to provide a theoretical basis for reclamation and ecological reconstruction in this area. The average well colour development (AWCD) absorption and Shannon-Wiener index values of soybean and maize rhizosphere microorganisms were higher than those of non-rhizosphere microorganisms, and the mean value of the fertilizer treatment was higher than that for CK. Principal component analysis shows the main carbon sources that impact the functional diversity of the soybean rhizosphere and non-rhizosphere soil communities are a-cyclodextrin, a-D-lactose, ß-methyl D-glucoside, and glucose-1-phosphate and L-phenylalanine, while those for the maize rhizosphere and non-rhizosphere soil communities are D-cellobiose, glucose-1-phosphate, ß-methyl D-glucoside, methyl pyruvate, D-galactosate gamma lactone, D-mannitol, N-acetyl-D-glucosamine, D-galactosalonic acid, and L-serine. The comprehensive utilization score of the non-rhizosphere soil carbon source in the maize season increased with respect to that in the soybean season, and the maximum increase was 1.09 under the OF treatment. Redundancy analysis showed that the soil nutrient factors driving the changes in the metabolic function diversity index values of the rhizosphere and non-rhizosphere soil microbial communities in the different crop seasons in the reclamation area differed, but they were all related to the soil organic matter and available phosphorus. This may explain why OF treatment is the most beneficial to soil fertility under the rotation system in mining reclamation areas.

## 1. Introduction

Land reclamation and ecological reconstruction can ease the tension of cultivated land, ensure food security, improve ecological environment. Soil quality is an important component in the reconstruction of ecosystems due to its physical, chemical and biological (nutrient) support for plant recolonization and establishment [1]. Soil microorganisms play an important role in soil biochemical processes, such as the decomposition of organic material, nutrient cycling, and the biotransformation of organic pollutants [2,3]. Soil microbial assessment can help lend insight into the processes that occur within a restored ecosystem [4]. Soil microbial diversity is a sensitive indicator of soil fertility, and soil metabolic activity is an important biological parameter associated with soil functions and is highly affected by agricultural practices [5]. It is crucial for soil nutrient recycling and utilization, energy transmission, ecosystem balance, and sustainable soil use in soil ecosystems [6,7,8,9]. Therefore, successful restoration relies on the regeneration of soil microbial communities [10,11,12,13]. Soil microbial communities are an important component of healthy soil [14]. One of the important functions of the microbial community is its ability to metabolize diverse carbon sources, as this feature is essential for organic matter turnover. Thus, the measurement of microbial diversity may provide important information regarding the function of microbial communities [15].

The rhizosphere refers to the microdomain environment within a few millimetres from the surface of the root axis and is the link connecting soil, plants, and microorganisms [16]. The rhizosphere, as a focus of microbial activity, plays an important role in microbial assembly because microbial-plant interactions and genetic exchanges are frequent in this area [17,18]. Rhizosphere microbial communities and root exudates promote plant growth by mobilizing nutrients and transforming organic matter in soil [19,20,13]. Microbial community compositions differ greatly due to the strongly selective environment of rhizospheres [21,22]. Bulk soil has relatively oligotrophic conditions, with low rates of nutrient transformation and microbial activity, unlike the more active rhizosphere environment [22]. However, it has also been reported that rhizosphere bacterial diversity is generally lower in the rhizosphere than in the bulk soil [23].

The soil in mining areas generally has the characteristics of low soil organic matter content, insufficient aggregates, and poor microbial activity [24,25,26]. Mining area reclamation efforts have been carried out in China for many years. There have been many reports on the impacts of planting vegetation and fertilization on the soil quality in mining reclamation areas [27,28,29]. However, little is known about the effects of different treatments on the carbon usage profiles of microbial communities from the point of view of plant rhizosphere and non-rhizosphere soil in crop rotation systems following mining reclamation.

Considering different crops and treatments, we hypothesized that (1) in different crop seasons, the rhizosphere soil microbial diversity index values would be higher than those of the non-rhizosphere soil, (2) the rhizosphere soil would have better comprehensive utilization of carbon sources than the non-rhizosphere soil, and (3) fertilizer would foster soil microbial carbon metabolism function, with the organic and inorganic fertilizer (OF) treatment having the best effect.

To test these hypotheses, the Biolog-ECO method was used to study the microbial carbon metabolism function of the plant rhizosphere and non-rhizosphere soil in the mining reclamation area of Gujiao Tunlan in Shanxi Province under a crop rotation system. The aim was to provide a theoretical basis for land reclamation and ecological reconstruction.

## 2. Materials & methods

### 2.1 Overview of the research area

The experimental area is located in the gangue discharge area of the Tunlan coal mine in the city of Gujiao, Shanxi Province, China (37°53′15″N, 112°6′42″E). The annual average temperature is 9.5 degrees, the average precipitation is 460 mm, the precipitation is concentrated in July and August, and the frost-free period is approximately 105 days, resulting in this area having a temperate continental monsoon climate. In 2012, after the discontinuation of gangue discharge, the gangue landfill area was covered with Malan loess soil. After natural subsidence for two years, the contents of heavy metals such as cadmium, lead and arsenic in the reclaimed soil were all lower than those of the two-grade standard of soil environmental quality (pH >7.5) (Table 1), indicating that the reclaimed soil could be used to grow crops. In 2014, farmland was reclaimed. In 2017 and 2018, leguminous and gramineous plants (soybean and maize, respectively) were planted. Four fertilization treatments were used in this experiment, namely, a control treatment (CK), inorganic fertilizer treatment (F), organic fertilizer treatment (O), and combined organic and inorganic fertilizer treatment (OF). Each experimental plot had a planting area of 0.33 ha, and the CK plots were fertilized at a rate of 0 kg/hm^2^, while the F, O, and OF treatments were fertilized at a rate of 150 kg/ha according to the same nitrogen content. The inorganic fertilizer used was a compound fertilizer (N:P_2_O_5_:K_2_O = 18:12:10) and was applied at a rate of 600 kg/ha, and the organic fertilizer was applied at a rate of 7500 kg/ha (organic matter content 53.48%, nitrogen content 2.2%). The combined organic and inorganic fertilizer treatment involved the application of the organic fertilizer at a rate of 3750 kg/ha and the compound fertilizer at a rate of 300 kg/ha. All fertilization methods involved artificial spraying.

**Table 1:** Actual values in the test area and soil environmental quality standards (national level II) (mg/kg).

Heavy metal	Zn	Cu	Cd	As	Pb
National standards	300	100	0.60	25	350
Actual Values	58	17	0.07	6	33

### 2.2 Soil sample collection

Precipitation and heat are abundant in July, and soybeans and maize grow quickly. On July 10, 2017, and July 5, 2018, the area was demarcated with 1 × 2 m plots on a diagonal line, and the shaking method was adopted [30]. Under the different fertilization treatments, 10 soybean and 3 maize plants of the same size were selected, and each treatment was replicated 3 times. Samples of rhizosphere soil were collected by excavating the plants and shaking off the large pieces of soil, and then the 0–5 mm layer of soil attached to the root system was gently brushed off and considered the rhizosphere soil sample. The surface soil between plant rows at 0–20 cm depth was considered as the non-rhizosphere soil, and samples were collected with the soil drilling method. Both rhizosphere and non-rhizosphere soil samples were collected in sterile plastic bags, which were then placed on ice and transported to the laboratory for analysis as soon as possible.

### 2.3 Soil chemical properties

The soil organic carbon (OC) content was determined using the potassium dichromate volumetric method [31]. Soil total nitrogen (TN) was measured using the Kjeldahl method [32]. Total (TP) and available phosphorus (AP) were extracted with H_2_SO_4_-HClO_4_ and sodium bicarbonate [33], respectively, and then determined with the molybdenum-blue method using an ultraviolet spectrophotometer [31]. Total (TK) and available potassium (AK) were dissolved with NaOH and extracted with ammonium acetate and then determined using the molybdenum-blue method with an ultraviolet spectrophotometer [31]. Alkaline nitrogen (AN) was determined with the alkaline hydrolysis diffusion method [31].

### 2.4 Biolog-EcoPlates experiment and data analysis

Biolog-EcoPlates^TM^ (Biolog, Inc., Hayward, CA, USA) were used to assess the soil microbial functional diversities based on the carbon source utilization patterns [34,35].

Ten grams of rhizosphere and non-rhizosphere soil was measured with an electronic scale, placed in a sterilized triangular bottle with glass beads with a concentration of 0.85% NaCl, and then fully oscillated at 250 r/min on a shaker for 30 min. After resting for approximately 20 min, the samples were diluted 10 times twice. Then, 150 μL of the soil inoculation solution was added to each of the 96 small wells in the Biolog-EcoPlates, which were then placed into an incubator at a constant temperature of 28°C. The Biolog analyser was used to read the absorption values of each hole every 24 h for 7 days.

The optical density for each well was calculated by subtracting the control well values for each plate from the optical density value of the well [34]. Microbial activity in each microplate was expressed using the average well colour development (AWCD) and calculated following the method of [34]. The 31 carbon sources were then classified as acids (n = 8), alcohols (n = 3), amino acids (n = 6), esters (n = 4), amines (n = 3), or sugars (n = 7) based on their chemical nature. Functional bacterial diversity was estimated using the Shannon-Wiener index (H’), Simpson’s index (D), and the evenness index (U), which can be calculated with the following formulas:

$$H’=‐\Sigma \;\mathrm{pi}\;*\;(ln\;\mathrm{pi});\;D=1‐\Sigma (\mathrm{Pi}{)}^{2};\;U=\sqrt{\left(\sum n_{i}^{2}\right)}$$

where p_i_ is the ratio between the optical density for each carbon source and the sum of all activities on the 31 substrates, and n_i_ is the relative absorbance of the ith hole.

### 2.5 Statistical analyses

MS-Excel and SPSS 22 were used to analyse the measured data with variance and principal component analysis (PCA). Significant differences among treatments were tested using one-way analysis of variance (ANOVA) followed by the LSD test. Redundancy analysis (RDA) was used to determine the relationship between the soil microbial diversity and nutrition with Canoco software, version 5.0.

## 3. Results

### 3.1 Dynamic change in the AWCD in the rhizosphere and non-rhizosphere soil in the mining reclamation area

**Fig 1:**
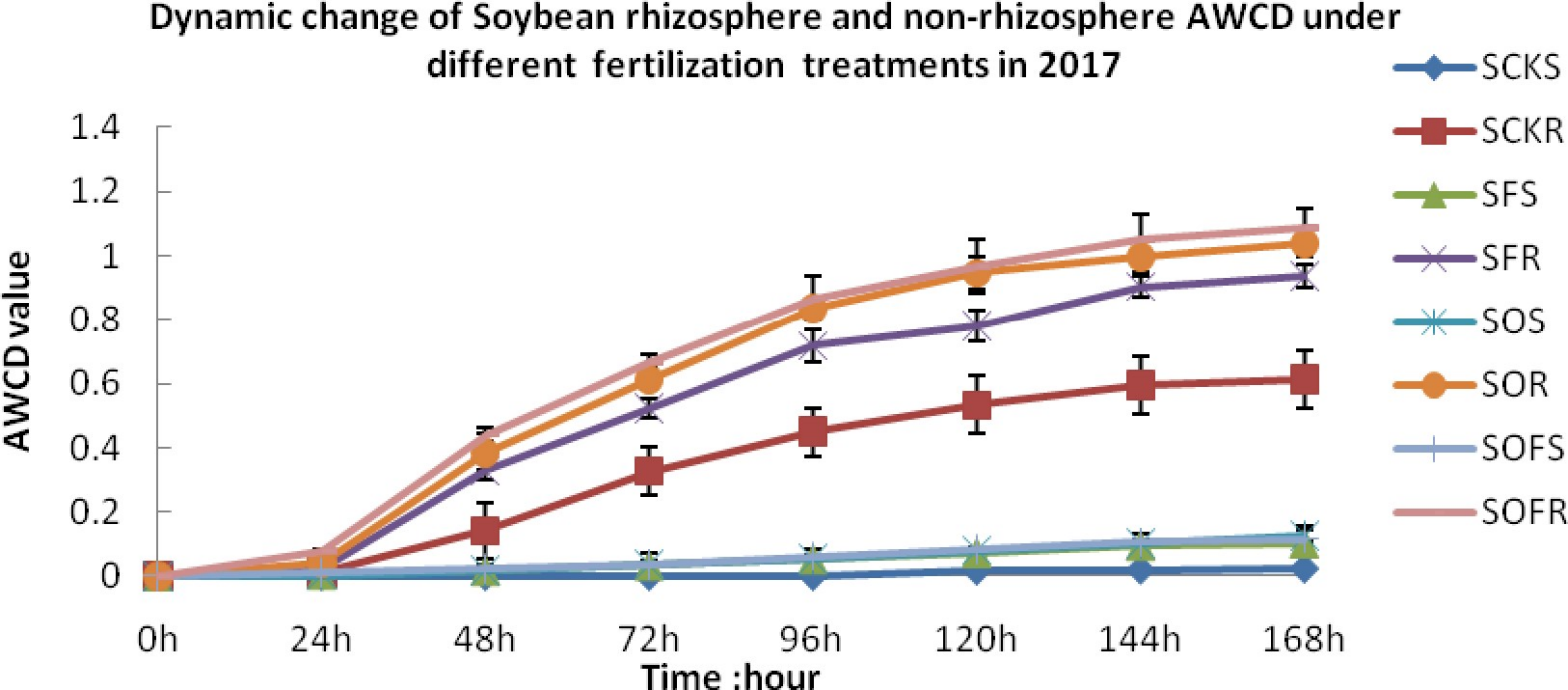
Dynamic change of soybean rhizosphere and non-rhizosphere soil in the mining reclamation area under fertilization treatments in rotation system. CK, F, O, OF represent different fertilizer treatments; the first S represents soybean; the second S represents non-rhizosphere soil. Figure represents three repeats.

In the soybean planting season in 2017, the AWCD value of the soybean rhizosphere and non-rhizosphere soil under the different fertilization treatments remained basically unchanged within 0–24 h but increased rapidly after 24 h. At all time points, the AWCD value of the rhizosphere soil under the different fertilization treatments was higher than that of the non-rhizosphere soil. The rhizosphere soil in the OF treatment had the highest AWCD value, and the lowest value was observed for the non-rhizosphere soil under the CK treatment. The order was SOFR > SOR > SFR > SCKR > SOS > SOFS > SFS > SCKS (Fig 1), where the first letter (S) represents soybean, the last letter (R or S) represents rhizosphere and non-rhizosphere, respectively, and the middle letters represent the treatment.

**Fig 2:**
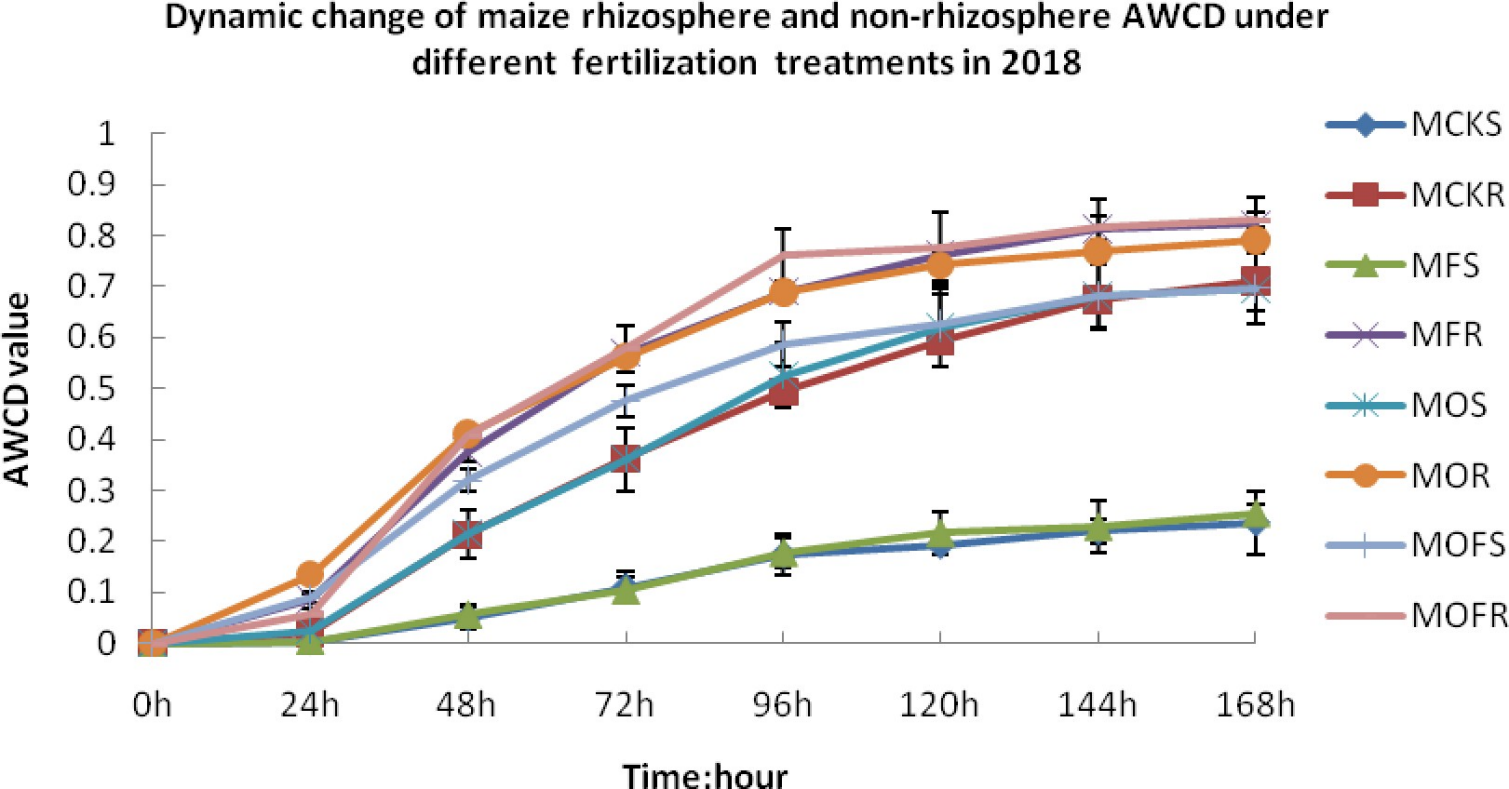
Dynamic change of maize rhizosphere and non-rhizosphere soil in the mining reclamation area under different fertilization treatments under rotation system. CK, F, O, OF represent different fertilizer treatments; the letter M represents maize; R represents rhizosphere soil; the letter S represents non-rhizosphere soil; Figure represents three repeats.

In the maize planting season in 2018, within 0–24 h, the value of the maize rhizosphere AWCD under the O treatment changed the most and was 0.13. The AWCD of the rhizosphere and non-rhizosphere soil under the F and CK treatments remained basically unchanged. After 24 h, the AWCD value increased rapidly under all treatments. After 96 h, the growth rate slowed. At all time points, the AWCD value of the maize rhizosphere soil was higher than the corresponding value of the non-rhizosphere soil. The time of 96 h represents the maximum moment of variability, and the order of the AWCD values was MOFR > MFR > MOR > MOFS > MOS > MCKR > MFS > MCKS (Fig 2).

Overall, the AWCD value of the crop rhizosphere was higher than the corresponding value for the non-rhizosphere soil for soil microorganisms in the mining reclamation area under the rotation system, and the microbial activity under the fertilization treatments was higher than that under the CK treatment. This demonstrates that fertilization can promote the formation and decomposition of root secretions of soybean and maize, improve soil microbiological activity, accelerate soil ripening in mining areas, and promote soil fertilization.

### 3.2 Microbial diversity characteristics of the rhizosphere and non-rhizosphere soils in the mining reclamation area

The functional diversity of soil microbial communities indicates the ability of microbial communities to use different types of carbon sources. Here, 96 h AWCD data were used to analyse and calculate indices of soil microbial diversity in the rhizosphere and non-rhizosphere soil associated with soybean and maize.

In the soybean season in 2017, the Shannon-Wiener index, evenness index, and Simpson’s index values of the rhizosphere soil microbial community under the different fertilization treatments were all significantly different from those of the non-rhizosphere soil microbial community (P<0.05). The highest mean Shannon-Wiener and Simpson’s index values were found for the soybean rhizosphere microorganisms under the OF treatment, and the smallest values were found under the O treatment. However, the non-rhizosphere soil microorganisms under the O treatment had the highest evenness index value, and there were no differences between the F and CK treatment evenness index values, although the F and CK values were significantly different from that under the OF treatment (P<0.05) (Table 2).

**Table 2:** Microbiological diversity index of maize rhizosphere and non-rhizosphere under different fertilization treatment in industrial reclamation area.

	Treatment	Shannon-wiener	Eveness	Simpson
2017 Soybean	SCKS	1.21±0.10e	0.45±0.12b	0.79±0.12ab
	SCKR	2.76±0.12b	0.25±0.01cd	0.94±0.00ab
	SFS	1.64±0.28d	0.49±0.05ab	0.75±0.05bc
	SFR	3.16±0.05a	0.22±0.01d	0.95±0.01a
	SOS	1.22±0.40de	0.61±0.19a	0.61±0.24c
	SOR	3.28±0.03a	0.20±0.00d	0.96±0.00a
	SOFS	1.98±0.20c	0.39±0.08bc	0.84±0.06ab
	SOFR	3.23±0.03a	0.21±0.00d	0.96±0.00a
2018 Maize	MCKS	2.45±0.24c	0.34±0.02a	0.89±0.01c
	MCKR	2.99±0.02a	0.24±0.00bc	0.94±0.00ab
	MFS	2.52±0.20c	0.33±0.05a	0.89±0.03c
	MFR	3.10±0.01a	0.23±0.00c	0.95±0.00ab
	MOS	2.98± 0.04a	0.24±0.01bc	0.94±0.00ab
	MOR	3.04± 0.01a	0.24±0.00bc	0.94±0.00ab
	MOFS	2.76±0.04b	0.27±0.00b	0.93±0.00b
	MOFR	3.19±0.03a	0.21±0.00c	0.96±0.00a

Different lowercase letters indicate significant difference at 5% level.

In the 2018 maize planting season, the microbial diversity index values showed no differences between the maize rhizosphere and non-rhizosphere soil under the O treatment (P > 0.05). However, under the other treatments, there were significant differences in the Shannon-Wiener index, evenness index and Simpson’s index values of the soil microorganism community (P<0.05) between the maize rhizosphere and non-rhizosphere soil. The mean Shannon-Wiener and Simpson’s index values for the maize rhizosphere microorganisms were the largest under the OF treatment, while those for the non-rhizosphere soil microorganisms were the smallest under the CK treatment, and these values showed no obvious difference from those under the F treatment (P > 0.05). The non-rhizosphere soil microorganisms under the CK treatment had the highest evenness index value, and the difference between the OF and O treatments was significant (P<0.05). Furthermore, the rhizosphere microorganisms under the OF treatment had the lowest evenness index value (Table 2).

### 3.3 Interannual variability in the utilization of various carbon sources by soil microorganisms under the rotation system

The rhizosphere and non-rhizosphere soil microorganisms showed varying utilization of the six types of carbon sources in the different crop seasons under the different fertilizer treatments.

The utilization of amino acids, amines and acids in the maize rhizosphere soil under the different fertilization treatments was lower than that shown by the soybean rhizosphere microorganisms, and the use of amino acids in the CK treatment decreased the most. However, the use of esters was higher than that shown by the soybean rhizosphere microorganisms, and the use of sugars and alcohols in the CK treatment was significantly higher than that in the other treatments (Table 3).

**Table 3:** Variation of the various carbon sources used in the non- rhizosphere soil under different fertilization treatments from 2017 to 2018.

		Sugar	Amino acid	esters	Alcohol	Amines	Acid
	SCKS	0.000±0c	0.004±0c	0.000±0d	0.000±0e	0.000±0c	0.001±0c
2017	MCKS	0.079±0.03c	0.261±0.15b	0.228±0.10c	0.119±0.04cd	0.402±0.20b	0.098±0.10bc
	SFS	0.045±0.04c	0.093±0.03c	0.001±0d	0.002±0e	0.005±0.01c	0.089±0.06c
	MFS	0.105±0.01c	0.250±0.04b	0.343±0.19c	0.143±0.08c	0.148±0.05c	0.130±0.03bc
	SOS	0.050±0.09c	0.020±0.01c	0.010±0.00d	0.026±0.01e	0.000±0c	0.138±0.10bc
2018	MOS	0.523±0.23b	0.548±0.13a	0.678±0.06b	0.589±0.06b	0.726±0.13a	0.339±0.12a
	SOFS	0.037±0.03c	0.049±0.01c	0.055±0.05d	0.048±0.05de	0.051±0.03c	0.106±0.05bc
	MOFS	0.831±0.06a	0.466±0.04a	0.897±0.15a	0.754±0.06a	0.591±0.08a	0.230±0.01a

Different lowercase letters indicate significant difference at 5% level.

The utilization of the different carbon source types by the non-rhizosphere soil microorganisms varied greatly. In 2018, it was significantly higher than that in 2017 across all treatments. The utilization in the fertilization treatments was significantly higher than that in the CK treatment, and the use of sugars, esters, and alcohols under the OF treatment was higher than that in the other treatments (Table 4).

**Table 4:** Variation of the various carbon sources used in rhizosphere soil under different fertilization treatments from 2017 to 2018.

		Sugar	Amino acid	Esters	Alcohol	Amines	Acid
	SCKR	0.074±0.05d	0.689±0.17abc	0.599±0.10d	0.388±0.14d	0.607±0.26bc	0.487±0.06bc
2017	MCKR	0.492±0.05c	0.394±0.02d	0.703±0.12cd	0.673±0.01abc	0.477±0.18c	0.406±0.04cd
	SFR	0.761±0.13b	0.806±0.16abc	0.831±0.20abc	0.566±0.14c	0.894±0.16a	0.549±0.15b
	MFR	0.879±0.07ab	0.607±0.03cd	0.925±0.09ab	0.807±0.02a	0.813±0.11ab	0.380±0.07cd
	SOR	0.895±0.08ab	0.835±0.15abc	0.778±0.06bcd	0.772±0.07ab	0.998±0.13a	0.776±0.00a
2018	MOR	0.861±0.03ab	0.666±0.08bc	0.979±0.04a	0.808±0.02a	0.847±0.01ab	0.307±0.01d
	SOFR	0.982±0.06a	0.914±0.19a	0.914±0.04ab	0.619±0.10bc	1.034±0.06a	0.741±0.06a
	MOFR	0.873±0.08ab	0.860±0.08ab	0.976±0.07a	0.606±0.06c	0.839±0.04ab	0.509±0.00bc

Different lowercase letters indicate significant difference at 5% level.

### 3.4 Key factors affecting the utilization of soil microbiological carbon sources under the rotation system in the mining reclamation area

Considering the 96 h AWCD value, 31 kinds of carbon sources were analysed by PCA to determine the key carbon sources that affect the microbial metabolism of rhizosphere and non-rhizosphere soil associated with soybean and maize, which is the basis for the rapid improvement of soil quality in mining reclamation areas.

**Fig 3:**
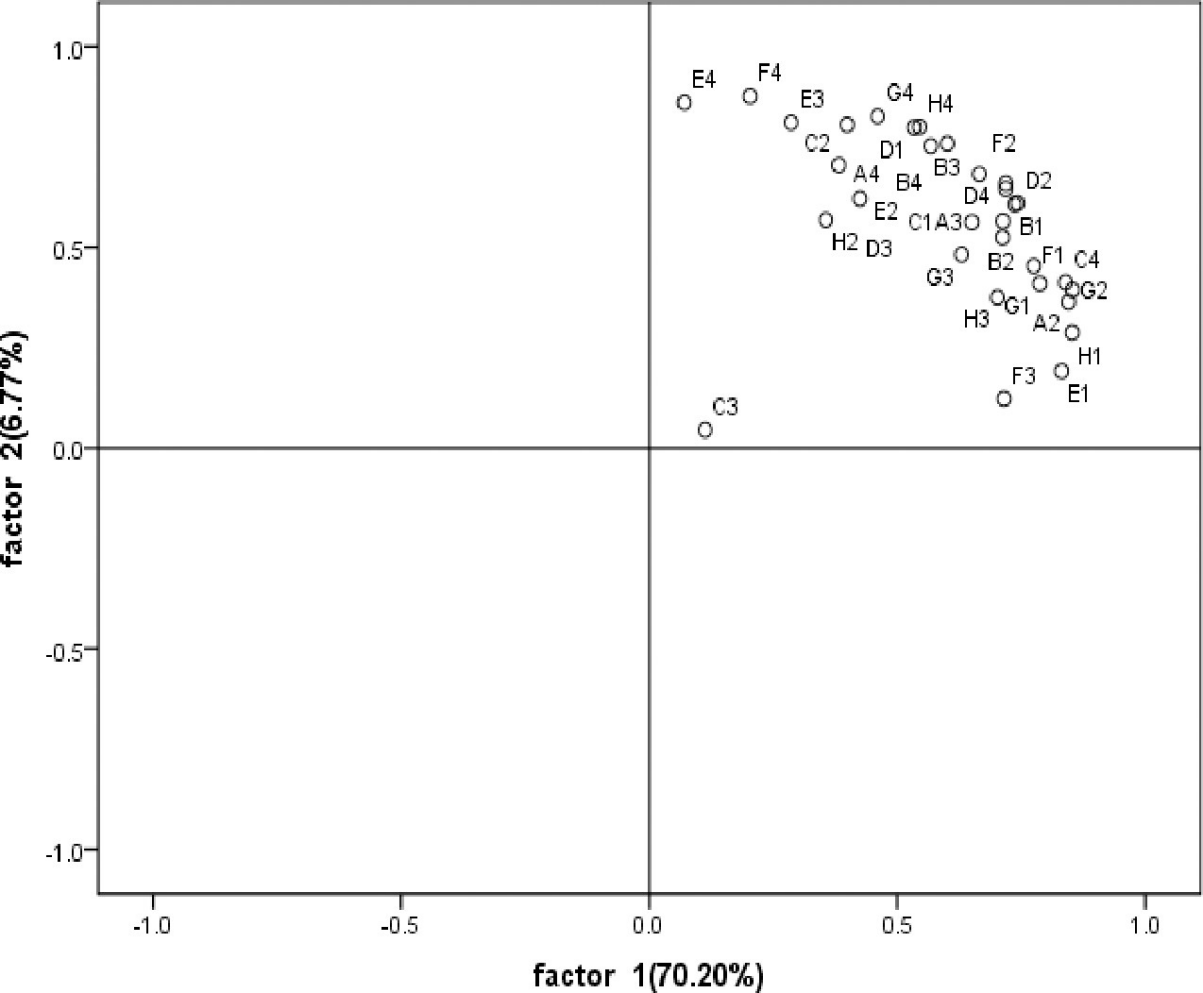
PCA about soybean rhizosphere and non-rhizosphere soil carbon sources utilization under different fertilization treatment in 2017. B1;C1;D1;E1;F1;G1;H1 represents respectively Methyl pyruvate; Twain 40; Twain 80; a-cyclodextrin; Glycogen; D-cellobiose; A-D-lactose.; A2;B2;C2;D2;E2;F2;G2;H2; represents respectively ß- Methyl D-Glucoside; D-xylose; I-gibberellin alcohol; D-mannitol; N-acetyl-D-glucosamine; D-glucosaminic acid; Glucose-1-phosphate; D, L-a-glycerol; A3;B3;C3;D3;E3;F3;G3;H3 represents respectively D-galactose gamma lactone; D-galacturonic acid; 2-hydroxybenzoic acid; 4-hydroxybenzoic acid; r -hydroxybutyric acid; Itaconic acid; a -butanoic acid; D-Malic Acid; A4;B4;C4;D4;E4;F4;G4;H4 represents respectively L-arginine; L-asparaginic acid; L-Phenylalanine; L–serine; L-threonine; Glycyl-L-glutamic acid; Phenylethylamine; Putrescine.

According to Fig 3, under the different fertilization treatments, soybean rhizosphere and non-rhizosphere soil microorganisms in the mining reclamation area are mainly related to E1 (a-cyclodextrin), H1 (a-D-lactose), A2 (ß-methyl D-glucoside), and G2 (glucose-1-phosphate), which are in the sugars group, and C4 (L-phenylalanine), which is in the amino acid group, on the first principal component. On the second principal component, the greatest positive correlation involves D1 (Twain 80) in the liquids group; E3 (r-hydroxybutyric acid) in the acids group; A4 (L-arginine), D4 (L-serine), E4 (L-threonine), and F4 (glycyl-L-glutamic acid) in the amino acids group, and G4 (phenylethylamine) and H4 (putrescine) in the amines group.

**Fig 4:**
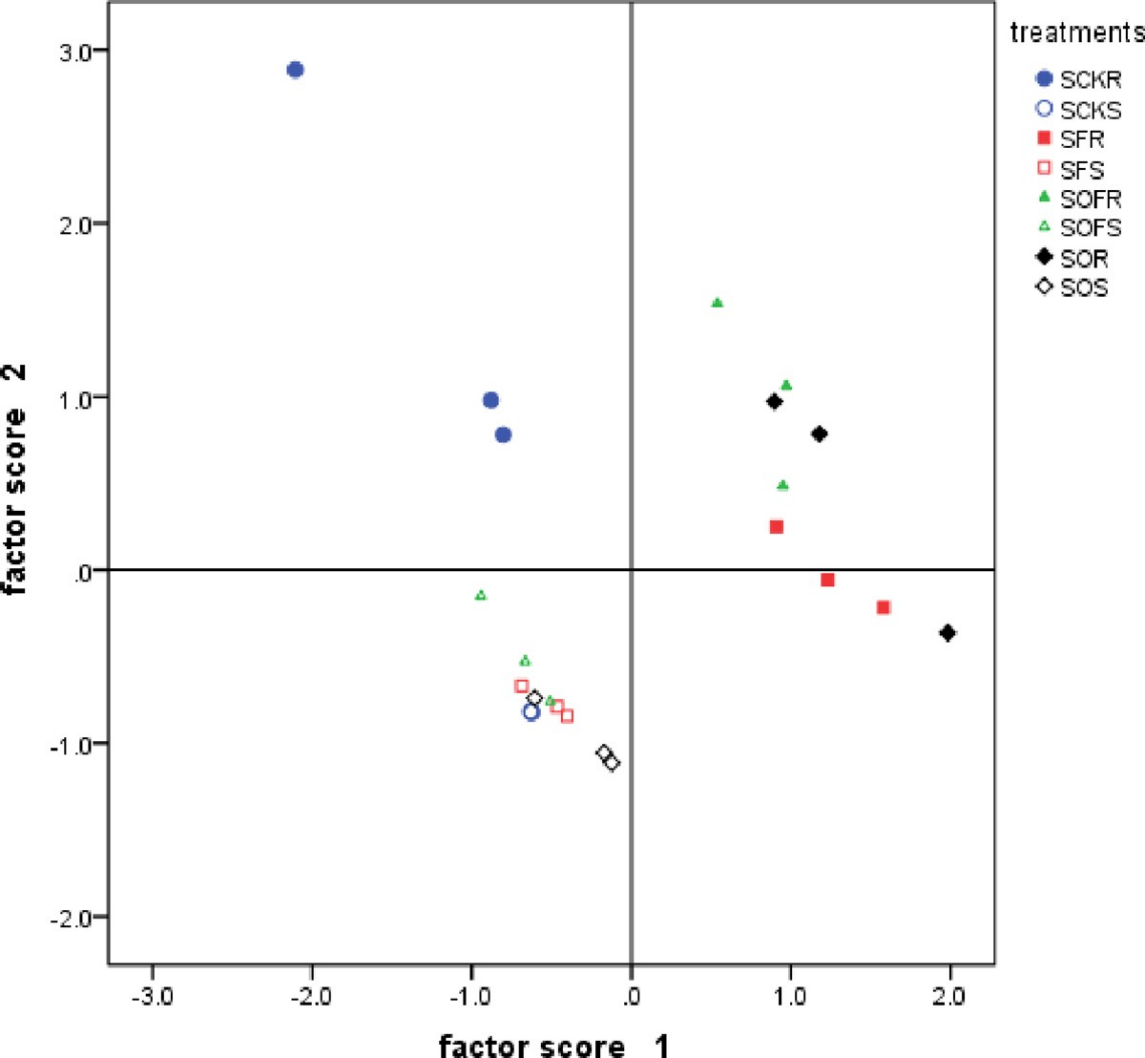
Factor score of soybean rhizosphere and non-rhizosphere soil carbon sources utilization under different fertilization treatments in 2017. CK, F, O, OF represent different fertilizer treatments; the first S represents soybean; the second S represents non-rhizosphere soil. Figure represents three repeats.

In Fig 4, non-rhizosphere soil is in the third quadrant, and rhizosphere soil is in the first, second, and fourth quadrants, indicating that there is a significant difference between non-rhizosphere and rhizosphere soil, and the SCKR treatment is located in the second quadrant. The results show that the fertilization treatments differed from the CK treatment in regard to the carbon metabolism ability of the soil microorganisms in the soybean rhizosphere.

**Fig 5:**
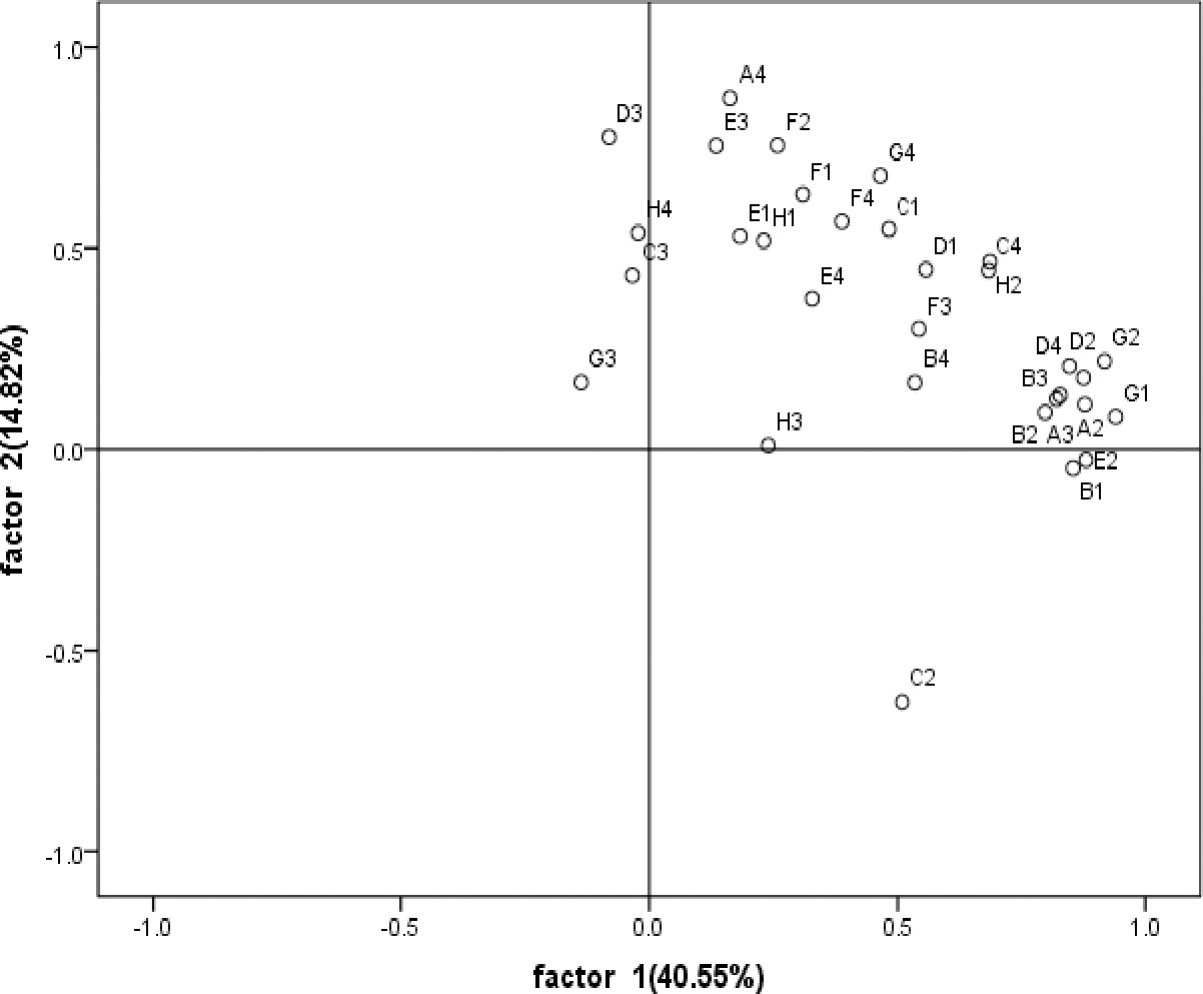
PCA about maize rhizosphere and non-rhizosphere soil carbon sources utilization under different fertilization treatments in 2018. B1;C1;D1;E1;F1;G1;H1 represents respectively Methyl pyruvate; Twain 40; Twain 80; a-cyclodextrin; Glycogen; D-cellobiose; A-D-lactose.; A2;B2;C2;D2;E2;F2;G2;H2; represents respectively ß- Methyl D-Glucoside; D-xylose; I-gibberellin alcohol; D-mannitol; N-acetyl-D-glucosamine; D-glucosaminic acid; Glucose-1-phosphate; D, L-a-glycerol; A3;B3;C3;D3;E3;F3;G3;H3 represents respectively D-galactose gamma lactone; D-galacturonic acid; 2-hydroxybenzoic acid; 4-hydroxybenzoic acid; r -hydroxybutyric acid; Itaconic acid; a -butanoic acid; D-Malic Acid; A4;B4;C4;D4;E4;F4;G4;H4 represents respectively L-arginine; L-asparaginic acid; L-Phenylalanine; L–serine; L-threonine; Glycyl-L-glutamic acid; Phenylethylamine; Putrescine.

According to Fig 5, in 2018, the maize rhizosphere and non-rhizosphere soil microorganisms in the mining area under the different fertilization treatments were mainly positively associated with G1 (D-cellobiose), G2 (glucose-1-phosphate), and A2 (ß-methyl D-glucoside) in the sugars group; B1 (methyl pyruvate) and A3 (D-galactosate gamma lactone) in the liquids group; D2 (D-mannitol) in the alcohols group; E2 (N-acetyl-D-glucosamine) in the amines group; B3 (D-galacturonic acid) in the acids group; and D4 (L-serine) in the amino acids group. The second principal component is mainly positively related to A4 (L-arginine) in the amino acids group and D3 (4-hydroxybenzoic acid), F2 (D-glucosaminic acid), and E3 (r-hydroxybutyric acid) in the acids group and negatively related to C2 (I-gibberellin alcohol) in the alcohols group.

**Fig 6:**
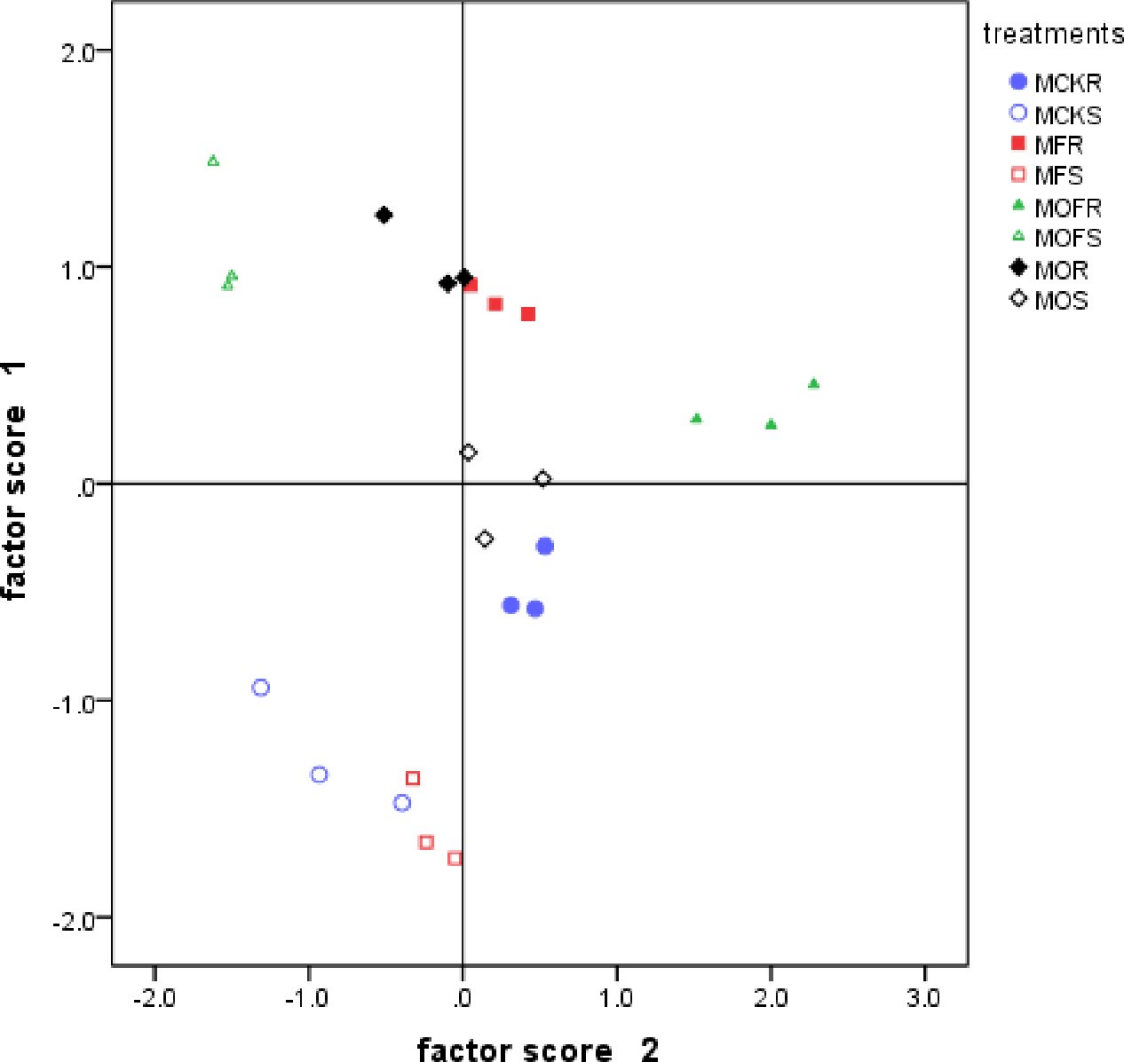
Factor score of maize rhizosphere and non-rhizosphere soil carbon sources utilization under different fertilization treatments in 2018. CK, F, O, OF represent different fertilizer treatments; the letter M represents maize; R represents rhizosphere soil; the letter S represents non-rhizosphere soil; Figure represents three repeats.

As seen from the factor score in Fig 6, the main component 1 separates the maize rhizosphere fertilization treatments from the MCK treatment. The MCKS and MFS treatments are located in the third quadrant, the MOFS treatment is in the second quadrant, and the MOS treatment is located near the origin. The results show that the MOFR treatment greatly differs from the other fertilization treatments and has a great influence on the comprehensive utilization of microbial carbon sources in the maize rhizosphere.

### 3.5 Capacity for the comprehensive utilization of carbon sources by microorganisms under the crop rotation system in the mining reclamation area

According to the principal component contribution rate and the component factor score of each treatment, the score values of the capacity for the comprehensive utilization of carbon sources of soybean and maize rhizosphere and non-rhizosphere microorganisms under the different fertilization treatments can be calculated. The comprehensive utilization capacity of microorganisms in the rhizosphere and non-rhizosphere soils under the fertilization treatments was higher than that under the CK treatment. The comprehensive utilization capacity of microorganisms in the non-rhizosphere soil under the OF treatment showed the greatest value increase (Table 5).

**Table 5:** Score value of comprehensive utilization of carbon source capacity in crop rhizosphere and non-rhizosphere under the crop rotation system from 2017 to 2018.

	CKR	FR	OR	OFR	CKS	FS	OS	OFS
2017 Soybean	-1.01	1.13	1.27	0.84	-0.65	-0.54	-0.36	-0.68
2018 Maize	-0.23	0.68	0.71	0.77	-1.15	-1.21	0.04	0.40
Increase	0.78	-0.45	-0.57	-0.07	-0.5	-0.67	0.40	1.09

### 3.6 RDA of microbial diversity indices and soil nutrients in the rhizosphere and non-rhizosphere soils under the rotation system

**Fig 7:**
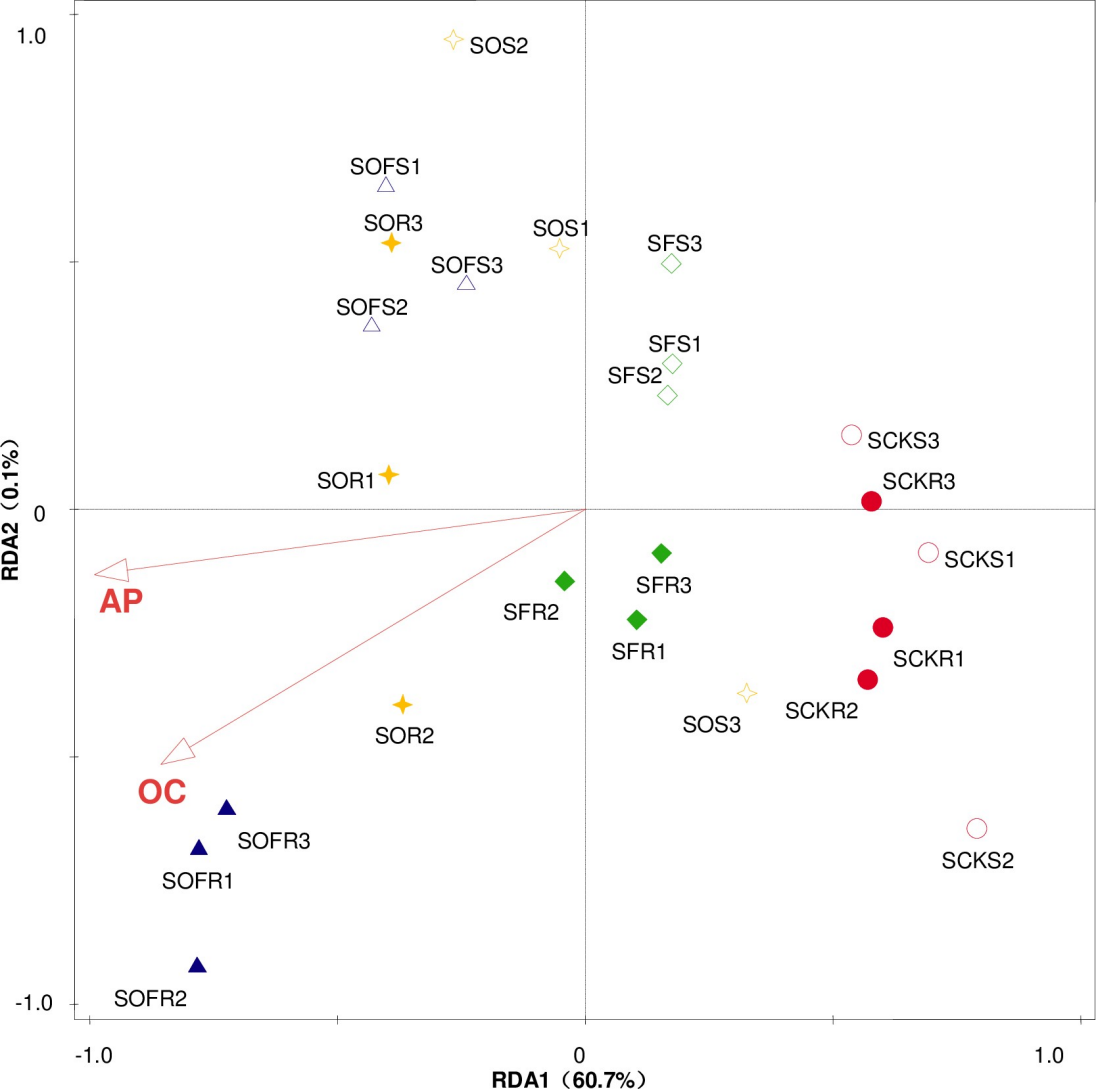
RDA in the soybean season. CK, F, O, OF represent different fertilizer treatments; the first S represents soybean; the second S represents non-rhizosphere soil; Figure represents three repeats.

**Fig 8:**
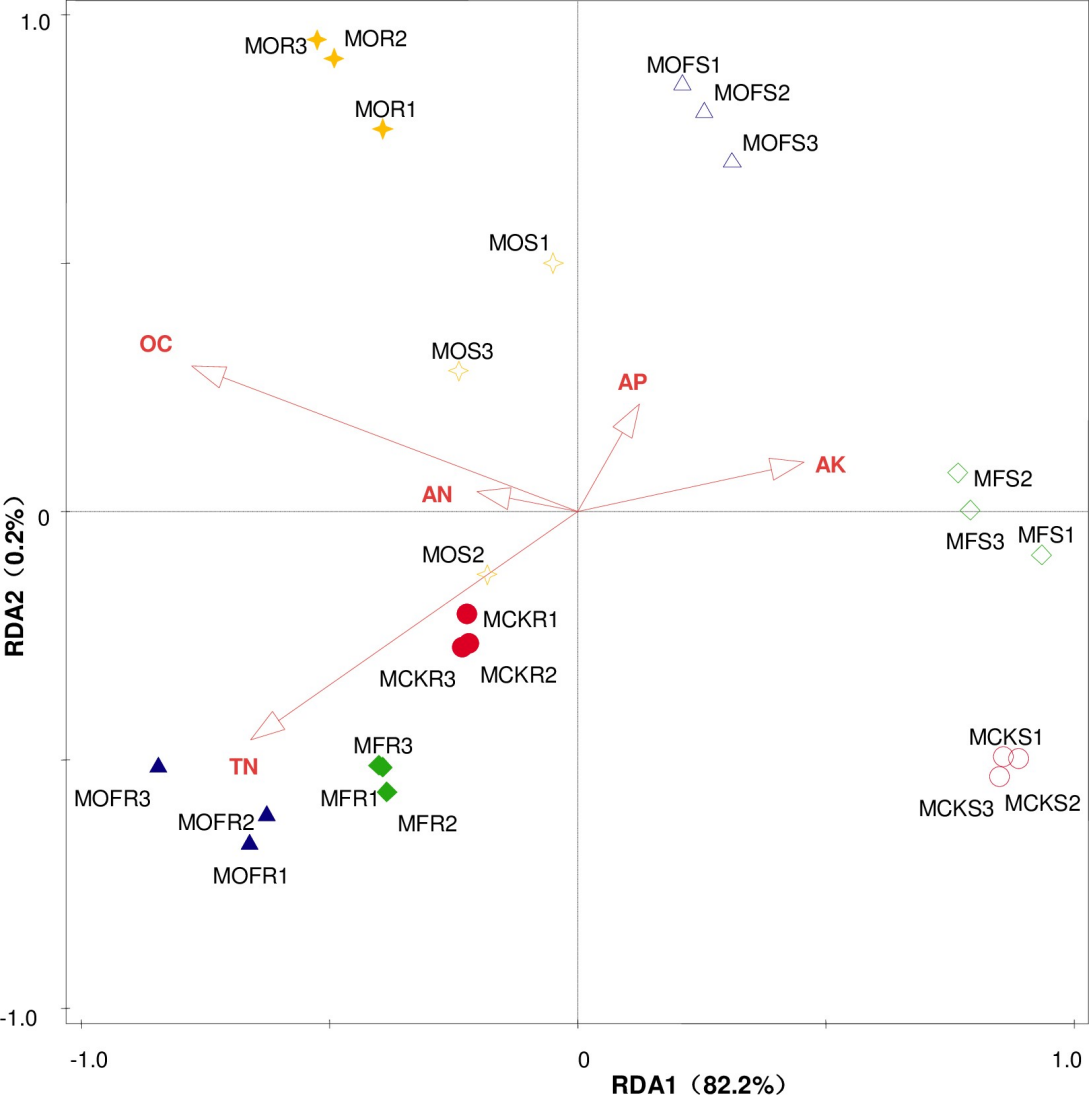
RDA in the maize season. CK, F, O, OF represent different fertilizer treatments; the letter M represents maize; R represents rhizosphere soil; the letter S represents non-rhizosphere soil; Figure represents three repeats.

Canoco 5.0 was used to perform an RDA of the rhizosphere and non-rhizosphere soil microbial diversity index values and organic matter, TN, TP, TK, AN, AP, and AK, and we found that in the maize season, the soil microbial diversity index values under the different treatments were effectively related to the soil organic matter, TN, AK, AN, and AP; in the soybean season, the microbial diversity index values for the rhizosphere and non-rhizosphere soil were only related to the soil organic matter and AP. The results showed that the factors affecting soil microbial diversity varied in the different crop seasons, but they were all related to the organic carbon and AP (Figs 7 and 8).

## 4. Discussion

Biolog-ECO can be used to obtain information on the overall activity and metabolic function of microbial communities, making up for the insufficiency of microbial community activity information provided by phospholipid fatty acid (PLFA), denaturing gradient gel electrophoresis (DGGE) and other molecular biology methods [36] because the substrates have representative members of the most frequently used compound groups [37]. Biolog-ECO is a valuable tool for the rapid and convenient screening of microbial functional abilities in rehabilitated mining landscapes. This method is sensitive and reproducible and yields information on important functional attributes of microbial communities [38]. In combination with PCA, Biolog-ECO can be used to explain the differences in the utilization patterns of soil microbial carbon sources. The different compositions and contents of different plant root secretions have varying effects on the growth and metabolism of soil microbial communities [39], and their effects on soil microbial communities thus differ [40].

### 4.1 Reclamation effect

**Fig 9:**
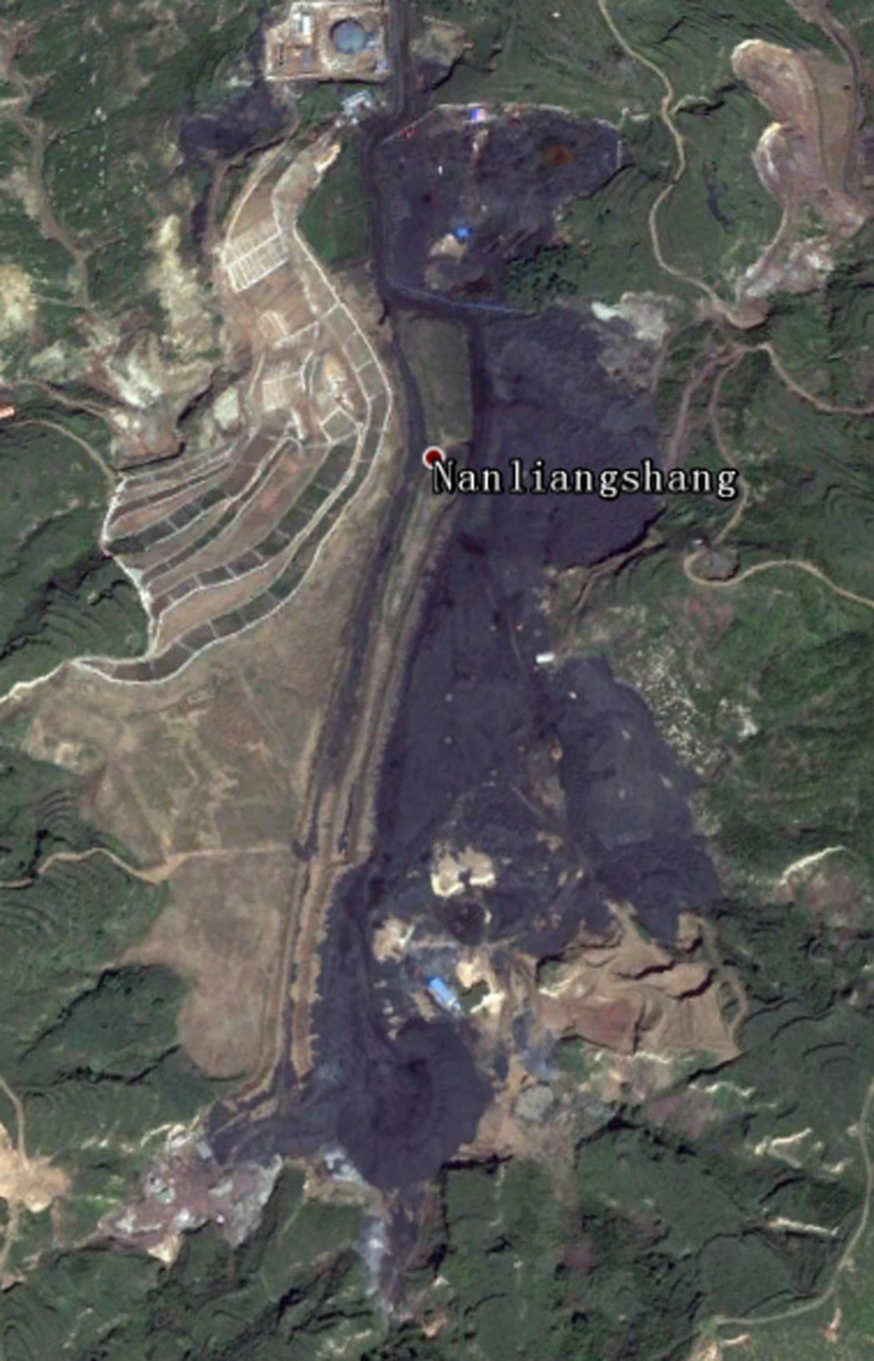
Landscape before reclamation in the researched area.

**Fig 10:**
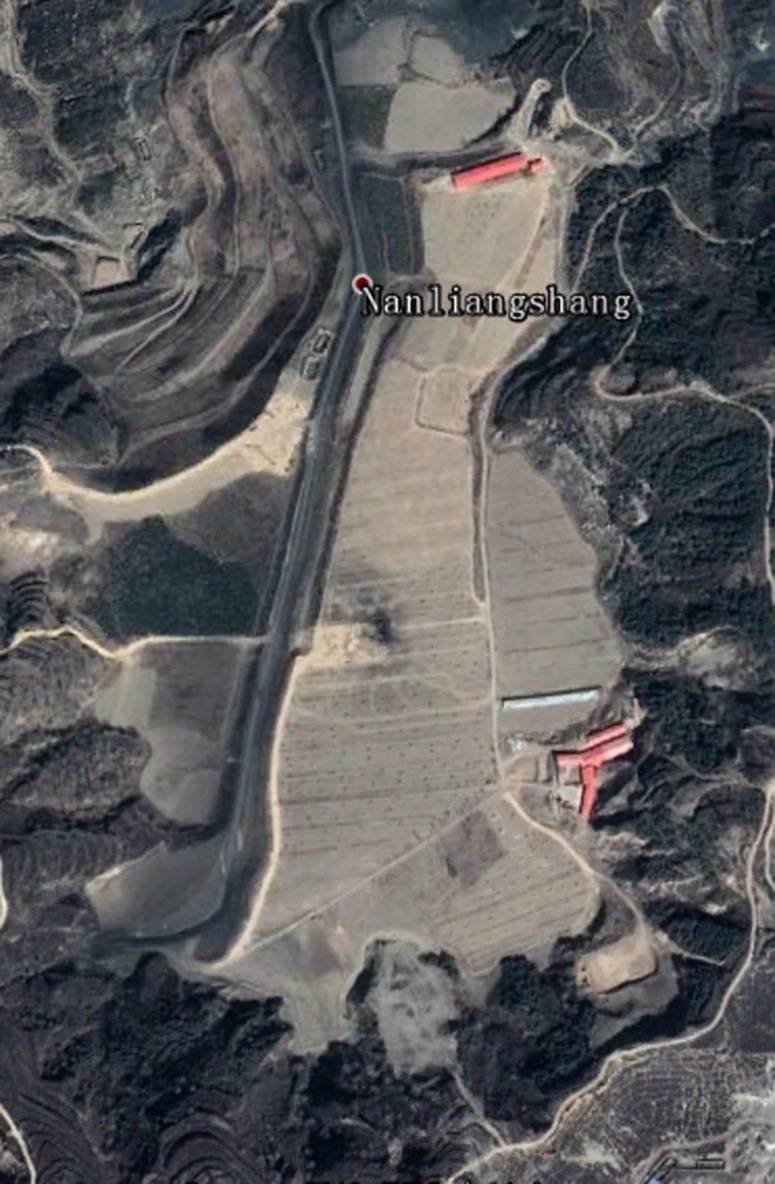
Landscape after reclamation in the researched area.

In the Loess Hills area, the gangue produced during the coal mining process is discarded into the ravines according to the production criteria. The landfill area formed by the gangue is shown in Fig 9. However, with the reclamation of the overlying new loess, the landfill reclamation area has been greatly changed, as shown in Fig 10.

The change is very significant in land productivity. The yield of the area was 0 kg/ha in 2013. However, soybean yield was 5350 kg/ha, and the aboveground biomass reached 10878 kg/ha under organic and inorganic fertilizer treatment in 2017. Maize yield was 7050 kg/ha and the aboveground biomass reached 13253 kg/ha under the same treatment in 2018.

The ecological restoration has also changed significantly at the same time. The vegetation coverage of the experimental area is increasing significantly. In 2013, the surface of the landfill area was bare and there was no biological growth, and the vegetation coverage was 0, while the vegetation coverage reached 25% in 2018.

In addition, after nearly five years of soil cultivation, the physical and chemical properties of reclaimed soil changed a lot. For example, compared with 2014, the soil bulk density decreased by 20%, the soil organic matter increased by 40%, the total nitrogen increased by 30% in 2018.

In summary, reclamation can not only improve the soil quality, increase the land productivity and grain yield [41] but also change the ecological landscape of the landfill area [42, 29].

### 4.2 Rhizosphere and non-rhizosphere soil microbial diversity

During the process of reclamation, plant growth plays a key role. The AWCD value is used to assess the total metabolic activity of soil microbes [34,43,44], with higher values indicating stronger microbial activity [45]. In general, microbial activity in the plant rhizosphere is higher than that in the non-rhizosphere. Plant roots release organic compounds, such as sugars, polysaccharides, amino acids, fatty acids, sterols, proteins, and secondary metabolites, in their exudates [46]. The non-rhizosphere soil is not (or is only slightly) affected by plant roots and root exudates, with a consequently lower level of microbial activity and soil fertility [43]. Similar results were also obtained in this study, i.e., the rhizosphere microbial activity was higher than the non-rhizosphere microbial activity in the period of vigorous growth in soybean and maize.

The Shannon-Wiener, Simpson’s and evenness indices are commonly used to characterize the diversity of soil microbial metabolic function [47]. Baudoin et al. [48] found that the largest difference in substrate (carbon source) utilization was between the bulk soil community and the maize rhizoplane community. Li et al., [49] showed that the Shannon index and evenness index values of the rhizosphere microbial community were higher than those of the non-rhizosphere microbial community in the podding and harvesting stages of soybean, while the Shannon-Wiener index and dominance index values of the rhizosphere microorganisms associated with soybean and maize were higher than those of the non-rhizosphere microorganisms, while the homogeneity index value was lower than that of the non-rhizosphere. The reason for this result may be that differences in rhizodeposit composition not only exist among plant types and soil types but also among stages of development in a single plant species [50,51,52,53]. These differences influence the density and diversity of rhizospheric microorganisms [54,55]. Therefore, the carbon source utilization ability also differs. The functional diversity of the microbial community in the reclamation area of coal gangue landfills during the soybean flowering period was studied in this experiment. The results show that the presence of organic substances such as carbohydrates and amino acids secreted by crop roots in different soil types during different growth stages might stimulate the growth of a specific microorganism, leading to an increase in the Simpson’s index value of the soil microorganisms, or inhibit the growth of certain microorganisms, leading to a decrease in the evenness index value of the soil microorganisms. For example, the PCA in this study showed that the utilization of microbial carbon sources in soybean rhizosphere and non-rhizosphere soil was correlated with L-phenylalanine in substrate amino acids, while that in maize rhizosphere and non-rhizosphere soil was correlated with L-serine in substrate amino acids.

### 4.3 Fertilization effects

Fertilization had a statistically significant impact on the soil microbial community and functional diversity [56]. Sarathchandra et al. [57] found that the functional diversity decreased when N was applied to pasture soils. Li et al. [58] reported that the microbial functional diversity was abundant in paddy soils subjected to long-term high fertilizer applications but decreased in vegetable farming soil subjected to excessive fertilizer applications. Some studies have reported that good fertilizer application management, including the use of appropriate types and quantities of fertilizers, can increase the microbial functional diversity to some extent [59,60,61].

To promote plant growth and fertilize the reclaimed soil, different fertilization measures were adopted in this experiment. The results show that the OF treatment improved the Shannon-Wiener index and Simpson’s index values of the soil microorganisms, but the evenness index value was reduced, which is in accordance with previous reports [63] but not with Yu et al. [55]. The results may be related to the characteristics of poor soil in the reclamation area. The OF treatment may lead to the homogenization of microbial communities in reclaimed soils in coal gangue landfill areas. The application of can significantly improve the carbon resource utilization of soil microorganisms in comparison to that in the CK treatment [62,63]. Because organic fertilizer can improve the carbon sources in the soil, inorganic fertilizer provides the necessary quick-acting nutrients for plant growth. Through the PCA, it was concluded that the comprehensive utilization score of soil microorganisms in regard to carbon sources increased positively under the combined application of organic and inorganic fertilizers, and the value increased the most, which further indicates that the combined application of organic and inorganic fertilizers had the most obvious effect on improving the quality of the reclaimed soil in this area. In addition, the RDA showed that organic matter and AP in the reclaimed soil were the main and common factors driving microbial diversity change in the soybean and maize rhizosphere and non-rhizosphere soil, which was similar to the results of Yu et al. [56].

## 5. Conclusion

(1) The results showed that microbial activity in the soybean and maize rhizosphere was higher than that in non-rhizosphere and that the Shannon index and Simpson’s index values of the functional diversity of the rhizosphere microbial community were higher than those for the non-rhizosphere microbial community, while the eveness index value was lower than that in the non-rhizosphere.

(2) Under the soybean-maize rotation system, the comprehensive utilization of carbon sources by non-rhizosphere soil microorganisms in the maize season was higher than that in the soybean season, and the combined application of organic and inorganic fertilizers could improve the functional diversity of the soil microbial communities.

(3) The main factors driving the functional diversity of the microbial communities in the soybean and maize rhizosphere and non-rhizosphere soils differed, and the common factors were organic matter and AP.

Because of the changes in natural environmental factors in the different growing seasons of plants, rhizosphere and non-rhizosphere microbial species may also vary. This may result in differences in the carbon sources that microorganisms may need in different planting seasons, which may cause metabolic function differences. Therefore, future research should deeply assess the difference between the rhizosphere and non-rhizosphere microorganism varieties in combination with species identification, which may reveal the mechanism of soil quality improvement in the mining reclamation area; this approach may require long-term experiments but can provide an improved theoretical basis for mining reclamation and ecological reconstruction work.

## Supporting information

S1 Data (ZIP)
